# Clinical, biological, echocardiographic and therapeutic determinants of the length of hospital stay of patients with Acute Heart Failure

**Published:** 2013-12-25

**Authors:** AC Nechita, V Enache, AM Stroi, RL Ploesteanu, C Delcea, CS Stamate

**Affiliations:** * 1st Internal Medicine and Cardiology Department, “Sfantul Pantelimon” Clinical Emergency Hospital Bucharest; ** „Carol Davila” University of Medicine and Pharmacy Bucharest; *** Neurology Department, Colentina Clinical Hospital Bucharest

**Keywords:** length of stay, LOS, acute heart failure, left ventricle ejection fraction, pulmonary artery acceleration time

## Abstract

Abstract

Hypothesis: The length of hospital stay (LOS) is a unanimously accepted measure of risk and treatment efficacy for in-patients.

Purpose: Our aim was to identify the parameters with predictive value for the LOS of patients with acute heart failure (AHF).

Methods: We analyzed 125 patients consecutively admitted to our clinic with a slight male predominance (54.4%) and a mean age of 71.54 years. Patients were divided into groups according to the clinical form at presentation and left ventricular function. Mean LOS was of 8.74 days.

Results: Patients with LVEF<30% had a significantly higher LOS compared to those with LVEF>30% (F(2)=6.54, p<0.05). The same difference was discovered for those who received inotropic support (p<0.001), i.v. loop diuretic>140mg (p<0.001) as well as for those with QRS>160ms (p<0.05) or LBBB. The linear regression equation exposed a single significant statistical model indicating that the need for vasopressor amines, mean diuretic dose and PAAT<90msec explain 56% of the variance of LOS F(3.46)=20.55, p<0.001. The highest contribution to the model was achieved by the need for vasopressor amines (β=0.66), with a unique contribution of 42% to the variance of the number of days of stay. The mean dose of diuretic had β=0.27 and a unique contribution to the model of 7.2%, followed by PAAT<90 msec with β=0.26 and a unique contribution to the model of 7%.

Conclusions: LOS is influenced by numerous parameters, some specific to certain clinical forms of AHF while others are independent, which is why evaluations on larger groups of patients are further needed.

## Introduction

Acute heart failure is one of the most important clinical syndromes because of the rising morbi-mortality rate, incidence and costs. It is the main cause of hospitalization in patients over 65 years old. In the United States it accounts for over one million admissions, six million days of hospitalization and costs estimated around 39.2 billion dollars [**1**-**3**]. In spite of all these estimates this syndrome has not been frequently studied in a systematic manner.

Recently, there have been discussions about the length of hospital stay and the costs that emerge from it, as well as about the appropriate moment to discharge the patient in safe conditions achieving the therapeutic goals. The pressure to reduce the number of days of hospitalization emphasizes the importance of prompt and aggressive treatment during the first hours from the admission and also the importance of therapeutic compliance in out-patients, because a large amount of admissions are, in fact, rehospitalizations in patients insufficiently compensated [**4**]. There are studies that report rates of readmission as high as 13% at 15 days after discharge and 25% at 30 days in the same population [**5**,**6**].

Other studies report that one third of patients over 70 years of age have hospitalization-associated disability, yet another important argument for the reduction of hospital stay [**7**], however under optimal conditions. That is the reason for seeking an algorithm of risk stratification in patients with AHF.

**Purpose**

We identified clinical, laboratory, echocardiographic and therapeutic parameters with predictive value for the duration of hospital stay in a group of patients with acute heart failure relevant for the determination of an algorithm of risk stratification and improvement of the disease prognosis.

## Methods

We analyzed 125 patients with AHF admitted consecutively in the Cardiology Department of “Sf. Pantelimon” Clinical Emergency Hospital Bucharest, Romania between June 2011 and March 2012.

We divided the group according to the clinical form of presentation and left ventricle ejection fraction (LVEF).

AHF classification according to the clinical form of presentation was made according to the recommendations of the European Society of Cardiology - Guidelines for the diagnosis and treatment of acute and chronic heart failure 2012 in six clinical categories: chronic decompensated HF, acute pulmonary edema (APE), hypertensive HF, cardiogenic shock, isolated right HF and HF associated to acute coronary syndrome (ACS). To avoid errors in the statistical analysis we excluded from the study all the cases in which more than 20% of the data were missing. Also, we did not take into consideration the last three clinical categories because of irrelevant statistical significance. We assessed arterial blood pressure (BP) on admission, atrial fibrillation, hemoglobin, sodium, creatinine clearance, NT pro BNP, echocardiographic parameters (EF, pulmonary artery acceleration time - PAAT, LV filling pattern), QRS duration, left bundle branch block (LBBB), the need for non-invasive mechanical ventilation, total dose of i.v loop diuretic and the use of inotropic and vasopressor drugs according to clinical forms at presentation.

For the statistical analysis we used SPSS 20.0. The impact of clinical and laboratory variables on short-term outcome was determined by using multiple regression equations while the differences between variables were calculated by using the analysis of variance for continuous variables and chi-square test for categorical variables.

## Results

**General Characteristics**

Of the 125 selected patients 54.4% were male and 46.6% female, with ages between 40 and 90 years old (71.54 ± 11.21 years). The most frequent form of presentation was chronically decompensated HF (58.4%), followed by APE (32%) and hypertensive HF (6.4%).

According to the ejection fraction, 11.2% of the patients had a LVEF<30% on admission, 31.2% had a LVEF between 30 - 40% and 57.6% a LVEF>40%. Decompensated CHF on admission was encountered in 76.9% of patients with LVEF <30% and only 54.4% of those with LVEF>40%. Of these, 8.8% had hypertensive HF on admission (χ2 (3) = 11.54, p <0.05).

APE was diagnosed in 23.1% of patients with LVEF<30%, 34.3% in those with LVEF between 30-40% and 33.8% in patients with LVEF>40%. Ischemic etiology was discovered in 76.9% of patients with LVEF<30%, 57% with LVEF between 30 - 40% and 25% of those with LVEF>40% (χ2 (2) = 17.95, p <0.001). 39.2% of all patients presented ischemic etiology of HF.

The main characteristics of the study group were assessed and compared according to the type of HF as well as the degree of ventricular dysfunction (Table 1, Table 2, Table 3).

**Table 1:**
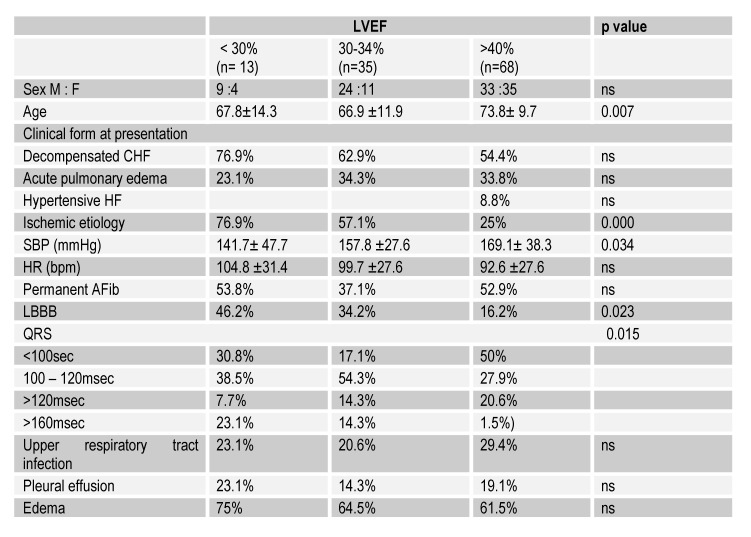
General data, clinical and electrocardiographic characteristics

**Table 2:**
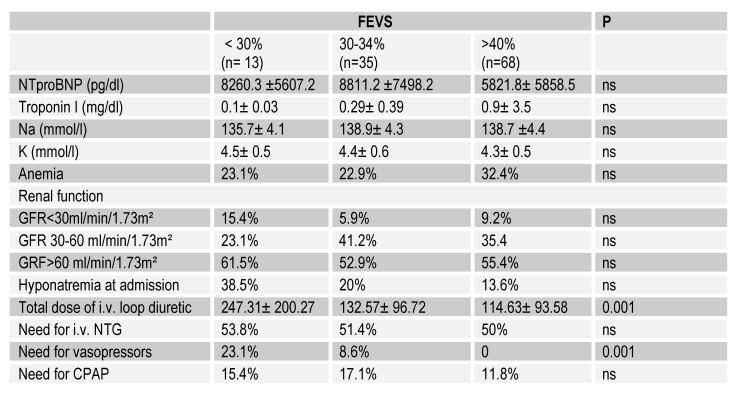
Laboratory parameters and classes of medication used in treating AHF

**Table 3:**
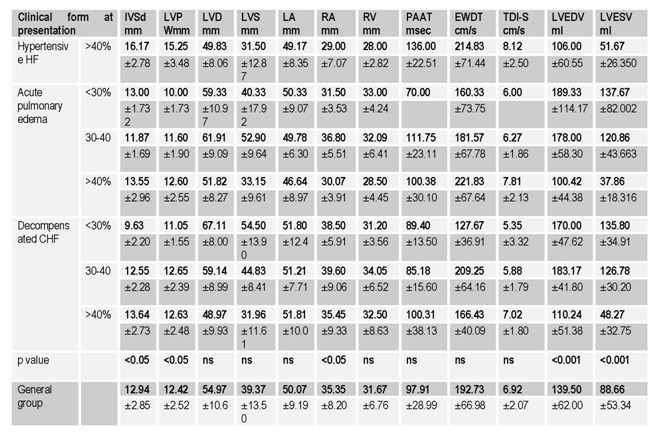
Echocardiographic characteristics according to the clinical form ar presentation and LVEF (mean±SD)

Concerning the precipitating factors, respiratory tract infections were documented in 26.4% of all patients. We found a significant association between the presence of decompensated CHF at presentation and respiratory infections on admission (χ²(1)= 4,51, p<0,05 ), diagnosis of decompensated CHF being 2.4 times more frequent in the presence of infection (OR = 2.4, 95% CI, 1.05 - 5.37). Attributable fraction to the infection associated with the diagnosis of decompensated CHF compared with other forms of presentation was 34.5%.

Echocardiography has recently emerged for the evaluation of patients with acute heart failure. It provides accessible, comprehensive and reproducible information. It often suggests the most likely mechanism for the hemodynamic imbalance in almost all situations, thus optimizing the therapeutic response. The most practical measurement of ventricular function is the LVEF. In our group, 38.4% of the patients had a preserved LVEF. Only 36.2% of patients with decompensated CHF and 44.7% of those presenting with APE had a LVEF> 50%, while the entire group of hypertensive HF had a LVEF> 50% (χ²(3)=11.54, p<0.05). Significant differences were also documented in terms of left ventricle end diastolic volume (LVEDV) (F (2) = 16.29, p <0.001) and left ventricle end systolic volume (LVESV) (F (2) = 30.96, p <0.001), with increased mean values in patients with LVEF <40% in decompensated CHF as well as APE. Tele-systolic diameters (F (2) = 24.21, p <0.001) as well as tele-diastolic ones (F (2) = 21.30, p <0.001) were significantly higher in patients with LVEF <40%, without significant differences according to the form of presentation, all these abnormalities representing common findings in patients with heart failure.

Regarding laboratory parameters, severely impaired renal function was associated to a greater extent with LVEF <30% (F (2) = 7.97, P <0.05), without significant differences according to the clinical form at presentation. Also, significant association between the presence of decompensated CHF and hyponatremia to presentation was discovered (χ²(1)= 4.98, p<0.05 ), the diagnosis of decompensated CHF being 3.1 times more frequent in the presence of hyponatremia (OR = 3.1, 95% CI 1.11 - 8.13), with attributable fraction to hyponatremia of 30.8%. As an outpatient therapeutic regimen, only 26.4% of the patients received maximal treatment while 27.2% were not previously prescribed cardiotropic treatment and 33.6% received a sub maximal, inconsistent or insufficient treatment.

**Average Length of Stay**

**Fig.1:**
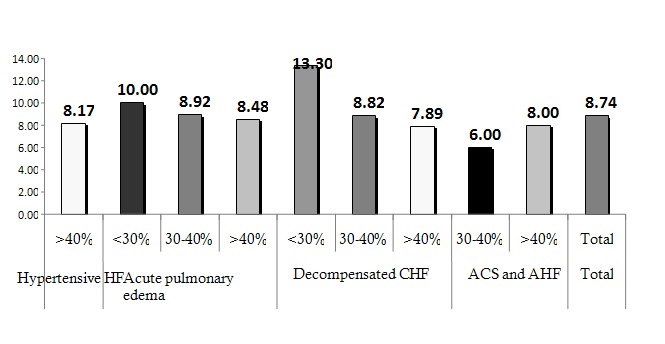
Mean length of stay according to the clinical form at presentation and LVEF

**Fig.2:**
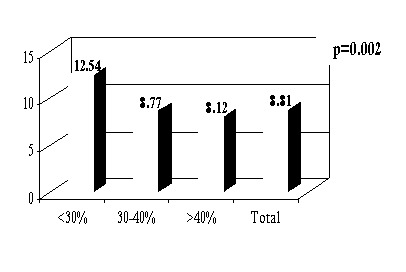
Mean length of stay according to the QRS duration and LVEF

The average LOS for the entire group of patients was of 8.74 days (**Fig. 1**), with an increased duration of hospitalization in patients with LVEF <<30% (12.54 ± 7.28) compared to those with LVEF> 30% (8.44 ± 3.53), regardless of the clinical form at presentation (F (2) = 6.54, p <0.05) (**Fig. 2**).

**Fig.3:**
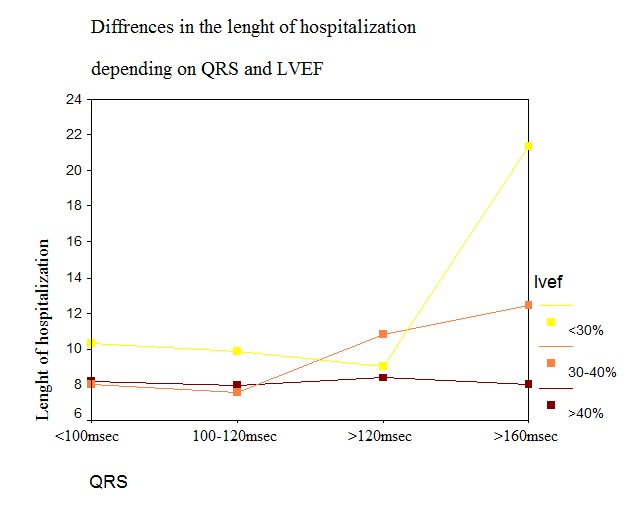
Mean length of stay according to QRS and LVEF

An increased duration of hospitalization was found in patients with QRS duration> 160msec (13.80 ± 7.88) both in the entire group (F (3) = 6.54, p <0.001) as well as according to LVEF (F (3) = 8.35, p <0.001) (**Fig. 3**), in patients with LBBB (10.23 ± 5.76) (F (1) = 5.37, p <0.05), in those who received inotropic support (19.17 ± 6.58) (F (1) = 59.93, p <0.001) and iv loop diuretic >140mg (10.04 ± 3.57) (F (1) = 9.08, p <0.05).

**Fig.4:**
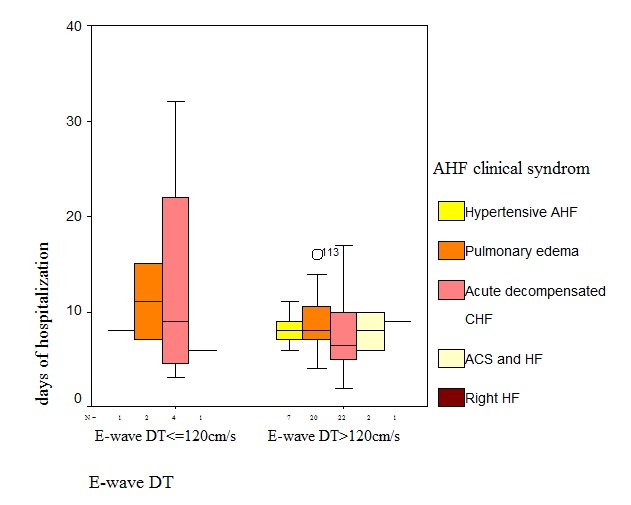
Mean length of stay according to the clinical form at presentation

An interesting finding was that there was an increase in the number of days of hospitalization in patients with decompensated CHF and PAAT<90ms (10.95 ± 6.77 days) as well as those with E wave deceleration time (EWDT)<120cm/s (13.25 ± 13.04 days), but without statistical significance (**Fig. 4**). Paradoxically, this is not true for the group with acute pulmonary edema, these parameters probably depending on the acute hemodynamic filling conditions and pre-existing pathological substrate.

**Prognostic factors**

The numerous categories of variables were analyzed to quantify the impact of various factors on the length of hospitalization in the analyzed group and several models were created by combining predictors. Multiple regression equation was applied and preliminary tests were conducted to verify the assumptions of linearity, normality, multicollinearity. We identified one statistically significant model which showed the need for vasopressor amines, the average dose of loop diuretic and PAAT<90 msec explained 56% of variance in the number of hospitalization days (F (3.46) = 20.55, p <0.001). The most important contribution to this model referred to the need for pressor amines (β = 0.66); it consisted of a unique contribution of 42% to the variance of the number of days of hospitalization. The mean dose of diuretic had a β = 0.27; its contribution to the model was 7.2%, followed by PAAT<90 msec, with β=0.26 and a contribution to the model of 7%.

## Discussion

Comparing the data encountered in the study group with the data published from the AHF registries regarding the general characteristics of the studied population there is some consistency. In the ADHERE registry, for example, the average age of the group was 75 years old, 52% of the patients being female, different to those enrolled in most clinical trials which enrolled patients with the tendency to be younger, predominantly male. Patients with preserved systolic function represent about half of the patients with AHF [**8**,**10**]. Other records suggest a mean age of 69.9 ± 12.5 years old, with 61% male predominance [9]. Regarding the clinical particularities, decompensated CHF is the most common clinical form of presentation. An average ejection fraction of 38% was documented, while a LVEF<30% was more commonly encountered in patients with decompensated CHF (34.6%) [**9**,**10**]. In our group, the mean age was 71.54 ± 11.21 years old, with 54.4% male predominance and 57.6% of patients with LVEF>40%. 

In EHFS-II the average length of hospitalization was 9 days for decompensated CHF, 10 days for APE, 8 days for hypertensive HF. There are authors who reported a mean hospital stay of 4-5 days [4] up to 13 days and even higher in patients with right heart failure [**11**]. In our study, the mean length of hospitalization was 8.74 ± 4.10 days. 

Administering inotropic agents could be associated with an increased risk of adverse outcomes, even with an increase in mortality, especially in patients with preserved LVEF [**12**]. 9% of patients enrolled in the ADHERE study received inotropic support with a significantly higher number of days of stay (12.9 days vs. 9.6 days) as well as mortality rate (19% vs. 14%).

More so, the patients with preserved systolic function, had a double mean length of stay (12.9% vs. 5.8%) with a mortality rate nine times higher (19% vs. 2%) [**13**]. In our group, the patients who needed inotropic support had an LVEF <40%, the strongest independent predictor for an increased length of hospitalization being the need for vasopressors. 

Another independent predictor for the duration of hospitalization in our group of patients was the necessary dose of i.v. diuretic. Although diuretic therapy rapidly improves the clinical presentation of patients with acute cardiac insufficiency, studies have been published linking the worsening of the prognosis of patients with cardiac insufficiency treated with high doses of diuretics [**14**,**15**] because of the consequences implicated in their administration. In the data analysis from the ADHERE study patients who received i.v. loop diuretics were noted to have an increased mean length of stay, without statistical significance. 

In another study where patients with CHF received resynchronization therapy, hyponatremia was noted to be an independent predictor for the worsening of heart failure after the implantation of the resynchronization device, alteration that could be partially explained by the rising levels of arginine vasopressin [**16**]. We found a significant association between decompensated CHF and hyponatremia on arrival with a larger number of days of hospitalization for these patients, however, without statistical significance.

Another independent predictor for the length of stay in our study was represented by the pulmonary artery acceleration time (PAAT<90ms). Pulmonary hypertension is classically considered a consequence or complication or the chronic cardiac insufficiency and probably a negative prognostic factor. Indirect criteria of pulmonary hypertension (PAAT<90 ms) and a restrictive echocardiographic filling pattern (TDE<120cm/s) were associated with an increase in the number of days of hospitalization in our group of patients. The fraction attributable to the presence of PAAT<90msec associated to the diagnosis of decompensated CHF, comparative to other forms of presentation was 40.7%. The same was not validated for the patients with APE probably because these parameters are dependent on the acute hemodynamic conditions of ventricular filling. 

## Conclusion

Our statistically significant conclusions, although concerning a small number of patients, enable further hypothesis testing on larger groups, including the development of a prognostic score in acute heart failure. We present our data in order to further discuss and study the parameters which were so far assessed to a lesser extent, other than the classical parameters, that might influence length of stay and short-term prognosis of patients with AHF.

The disclosed analysis may be important for a better understanding of this pathology and further development of new methods of prevention and effective treatment. On the other hand, its assessment raises a prognostic value, helping us stratify patients, individualize the therapeutic regimen and allow the definition of subgroups of patients that may benefit from a particular therapy. The beneficial economic impact is induced by the results that could allow optimization of evaluation and treatment of patients with heart failure.
